# Willingness-To-Accept Pharmaceutical Retail Inconvenience: Evidence from a Contingent Choice Experiment

**DOI:** 10.1371/journal.pone.0126790

**Published:** 2015-05-29

**Authors:** Keith Finlay, Charles Stoecker, Scott Cunningham

**Affiliations:** 1 Department of Economics, Tulane University, New Orleans, LA, USA; 2 Department of Global Health Management and Policy, Tulane University School of Public Health and Tropical Medicine, New Orleans, LA, USA; 3 Department of Economics, Baylor University, Waco, TX, USA; The University of Kansas Medical Center, USA

## Abstract

**Objectives:**

Restrictions on retail purchases of pseudoephedrine are one regulatory approach to reduce the social costs of methamphetamine production and use, but may impose costs on legitimate users of nasal decongestants. This is the first study to evaluate the costs of restricting access to medications on consumer welfare. Our objective was to measure the inconvenience cost consumers place on restrictions for cold medication purchases including identification requirements, purchase limits, over-the-counter availability, prescription requirements, and the active ingredient.

**Methods:**

We conducted a contingent choice experiment with Amazon Mechanical Turk workers that presented participants with randomized, hypothetical product prices and combinations of restrictions that reflect the range of public policies. We used a conditional logit model to calculate willingness-to-accept each restriction.

**Results:**

Respondents’ willingness-to-accept prescription requirements was $14.17 ($9.76–$18.58) and behind-the-counter restrictions was $9.68 ($7.03–$12.33) per box of pseudoephedrine product. Participants were willing to pay $4.09 ($1.66–$6.52) per box to purchase pseudoephedrine-based products over phenylephrine-based products.

**Conclusions:**

Restricting access to medicines as a means of reducing the social costs of non-medical use can imply large inconvenience costs for legitimate consumers. These results are relevant to discussions of retail access restrictions on other medications.

## Introduction

Methamphetamine (meth) use is a significant social ill that has been linked to personal consequences ranging from dropping out of school [1] to heart attacks [2], and social consequences from violent behavior [3] to increased infectious disease transmission [4]. Methamphetamine can be easily synthesized in small batches from precursor ingredients found in widely available nasal decongestant medicines containing pseudoephedrine (pseudo) [5]. Due to these social costs, policies have been enacted since the early 1990s to curtail domestic access to pseudo in an effort to reduce domestic meth synthesis and, ultimately, consumption via the policy’s effect on meth availability and prices.

Increased sales of pseudo have been linked with increased meth production [6], so regulations have placed legal constraints on retail sales at pharmacies. Retail pharmacy regulations that restrict consumer purchase of pseudo are usually bundled constraints along numerous dimensions. For instance, the federal Combat Methamphetamine Epidemic Act of 2005 restricted retail purchases to no more than 9 grams of pseudo per month, required consumers to present proof of identification at point of purchase, and moved pseudo behind the counter. Oregon and Mississippi enacted “prescription-only” laws in 2006 and 2010, respectively, that further restricted access. At least a dozen states have passed laws that require pharmacies to record all identifying information from consumer purchases of pseudo into a centralized database for real-time tracking.

Each of these constraints impacts legal consumers of pseudo by raising the marginal cost of purchase. Requiring identification may impose direct costs, and potentially cause equity issues, since obtaining official identification may be more burdensome to lower-income consumers [7]. Purchase limits may impose inconvenience costs such as additional time or costs for travel. Prescription requirements include direct costs, travel costs, time costs to both patient and prescribing doctor, and can be substantial [8]. A full evaluation of these interventions would compare the benefits of each of these strategies, including potentially reduced social costs of methamphetamine production and use, with the harms.

Previous work has speculated about the existence of substantial costs of access restrictions to medications in general to consumer welfare [9, 10]. Studies have explored the consequences of interactions of prescription requirements and insurance status on consumer costs [11, 12]. This is the first study, however, to measure the inconvenience burden to consumers associated with medication access restrictions.

In this paper, we aim to identify the costs to consumers of identification requirements, purchase limits, behind-the-counter requirements, or prescription requirements. We use a contingent choice experiment and model responses with a conditional logit to calculate willingness-to-accept each restriction.

## Methods

### 0.1 The choice experiment

We asked 2,000 survey participants to suppose they were interested in buying cold medicine. After a brief orientation to the two principal active ingredients in nasal decongestants (included in S1 Pseudo Orientation), each participant was asked to choose an option for treating a cold. Respondents were presented with a set of 4 choices: 2 pseudo-based product options with different restrictions, a phenylephrine-based (phenyl) product with no restrictions, or a no-purchase option. We included the phenyl alternative to pseudo to capture some measure of how willing consumers would be to substitute away from pseudo medications to possibly less effective and less restricted phenyl medications. Each participant was presented with 10 randomly ordered sets of 4 randomly ordered alternatives. We chose 4 alternatives as this is close to an optimal choice set size [15]. The Tulane University IRB Board determined this study was exempt from oversight.

Phenyl decongestants (such as Sudafed Pressure and Pain) are widely available in retail pharmacies and marketed as an effective over-the-counter alternative to pseudo-based nasal decongestants (such as Sudafed 12-Hour). On a molecular level, these chemicals differ in how much is metabolized by the body and which receptors are affected. Only 38% of phenyl is absorbed for effective use by the body compared with 100% for pseudo. Pseudo is a stimulant that releases adrenaline, whereas phenyl does not have this effect. Whereas the efficacy of pseudo as a nasal decongestant is supported by numerous controlled trials, there is little evidence that oral phenyl performs better than placebo [13]. See Eccles [14] for a detailed comparison between pseudo- and phenyl-based nasal decongestants.

The pseudo options were presented with restrictions including an identification requirement at the point of purchase, a purchase limit, a prescription requirement, or availability restricted to behind-the-counter. In order to reduce the number of options presented, we used a D-efficiency criterion to construct our alternatives. For each alternative there were 5 attributes: active ingredient (psuedo, phenyl, or no purchase), price (random, random with a discount), ID requirement (yes, no), purchase limit (yes, no), and purchase experience (easy to buy as Tylenol, behind the counter, prescription). This left us with 72 possible alternatives. In order to administer the choice experiment that minimized the variance of estimators we selected the set of alternatives that maximized D-efficiency by choosing the set with that maximizesd the determinant of the information matrix [16]. We eliminated illogical combinations of restrictions (such as prescription requirements that do not also include an ID requirement) and dominated alternatives, as suggested by [17] and [18], (such as phenyl with purchase restrictions). This procedure left 11 potential alternatives. The 7 salient alternatives shown in Table 1 maximized the determinant of the information matrix. The alternatives we presented to respondents were 1) pseudo requiring identification and prescription, 2) pseudo requiring identification and with a 1 box per month limit, 3) pseudo with a 1 box per month limit, 4) pseudo requiring identification, 5) pseudo without restrictions, 6) phenyl without restrictions, and 7) no purchase.

**Table 1:** Contingent choice alternatives.

Alt. (1)	Active ingredient (2)	ID required (3)	Purchase limit (4)	Purchase experience (5)
1	Pseudo.	×	set by doctor	R_x_
2	Pseudo.	×	1 box/month	BTC
3	Pseudo.		1 box/month	BTC
4	Pseudo.		no limit	OTC
5	Pseudo.	×	no limit	BTC
6	Phenyl.		no limit	OTC
7	[No purchase]			

Notes: Each choice set consisted of a pair of pseudoephedrine alternatives along with the phenylephrine and no-buy alternatives.

**Fig 1:**
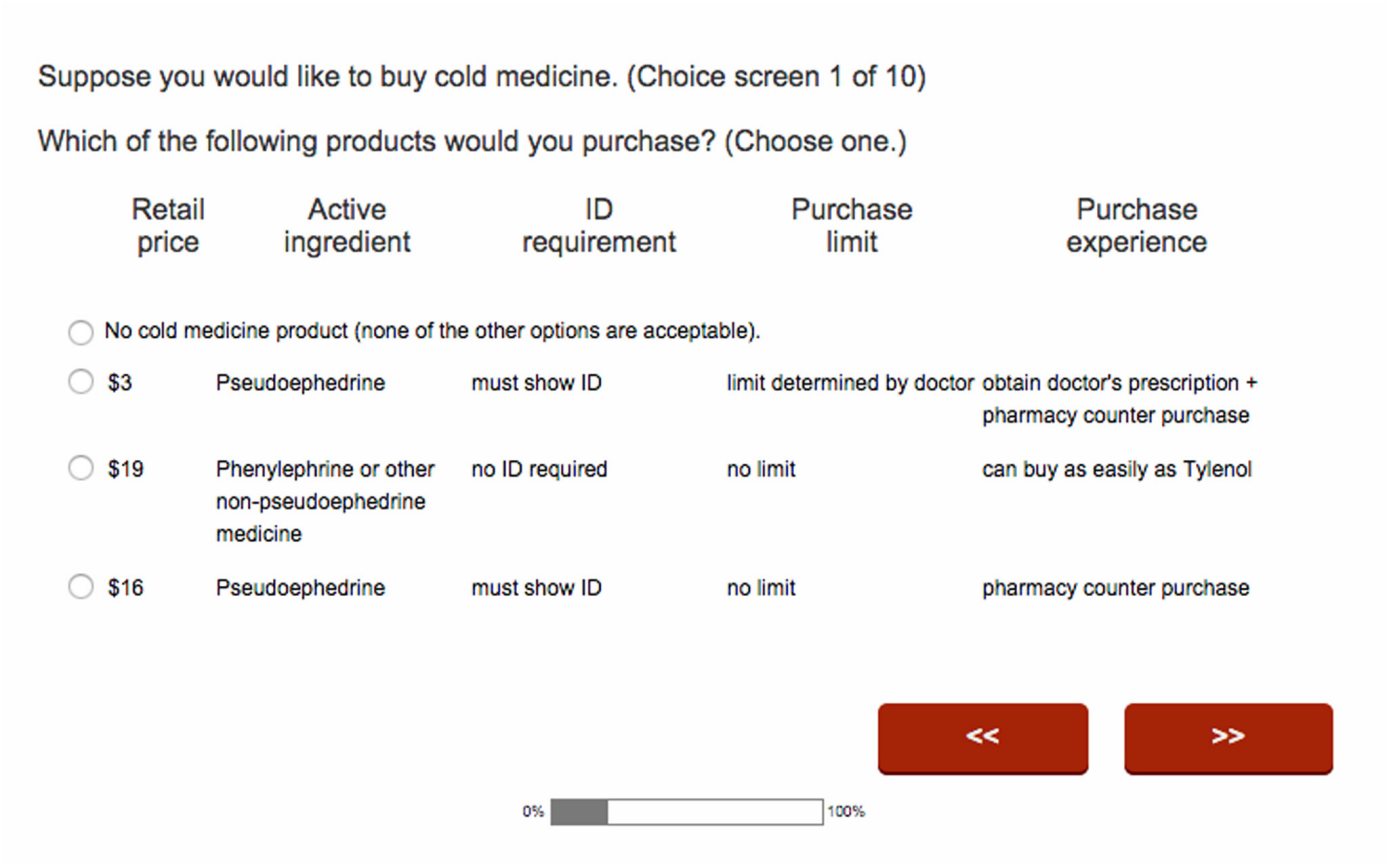
Example choice set screen from contingent choice survey.

Each of the purchased options was presented with an out-of-pocket price, which we modeled as a draw from a base price distribution and accompanying discount factor. The base prices were drawn from a uniform distribution between $6 and $30 independently for each option. To account for the compensating differentials buyers would require for potential restrictions, we further applied a discount to pseudo. The discount was drawn from a uniform distribution that was between $1 and $1 less than the base price. For example, a pseudo option with a randomized base price of $13 could have a discount of $4. When users were presented with a pseudo purchase option without restriction we added a number between $1 and $1 less than the base price to the pseudo option to account for compensation buyers would require when purchasing the option with the potentially less desirable active ingredient. The respondent was only presented with the final price, in this case $9. Fig 1 shows an example choice screen used in the experiment. At the start of the survey we asked respondents what their out-of-pocket expense was when they went to the doctor’s office, for use in later analysis.

We used this Choice Experiment (CE) method instead of directly asking participants to report monetary values for each restriction. Direct question surveys can lead to respondents valuing whole items instead of the attributes of the choices [19]. The CE method allows us to calculate a value for each specific attribute. It is also efficient at drawing information from respondents by allowing us to elicit values for several potential permutations of precursor restrictions in each test. For a thorough review of the CE method in health applications, see [20], and for detailed descriptions of choice experiment design see, [21] and [22].

### 0.2 Mechanical Turk

We administered our survey online using Amazon Mechanical Turk in November 2014. Mechanical Turk provides an online marketplace that connects occasional Internet workers (here survey respondents) with labor purchasers. We paid each respondent $1, which is slightly higher than the typical compensation [23]. Results from field and laboratory surveys and experiments have been replicated using Mechanical Turk [24]. Survey responses from Mechanical Turk are typically more representative than from other online survey mechanisms or convenience samples of college students [25]. We further refined the representativeness of our analysis by calculating survey weights for our respondents [26] to replicate the characteristics of the general population from the 2012 5-year American Community Survey (ACS). The ACS is administered annual by the U.S. Census Bureau to 3 million households. The ACS 5-year release contains the most recent 5-years of data. We balanced respondents in our sample with those in the ACS according to gender, race, highest education completed, number in the household, marital status, census division, and household income. The standardized differences in these attributes between our sample and the ACS are shown in Table 2. After weighting, the standardized difference between our survey respondents and the population average from the ACS was less than 0.00001 for each category of observable characteristics.

**Table 2:** Means and standard errors of weighting variables, ACS reference sample, unweighted MTurk sample, weighted MTurk sample.

Variables	ACS sample (1)	Unweighted MTurk sample (2)	Weighted MTurk sample (3)	Standardized diff.: (3)−(1) (4)
Female	0.519	0.458	0.519	−0.00000
Asian	0.057	0.090	0.057	0.00000
Black	0.122	0.088	0.122	0.00000
White	0.779	0.830	0.779	0.00000
Hispanic	0.143	0.100	0.143	−0.00000
Household size: 1	0.139	0.200	0.139	−0.00000
Household size: 2	0.336	0.303	0.336	−0.00000
Household size: 3	0.194	0.229	0.194	0.00000
Household size: 4	0.171	0.160	0.171	0.00000
Household size: 5	0.088	0.070	0.088	0.00000
Household size: 6+	0.072	0.037	0.072	−0.00000
Up to high school graduate	0.427	0.112	0.427	−0.00000
Some college	0.233	0.281	0.233	0.00000
2-year college degree	0.074	0.121	0.074	0.00000
4-year college degree	0.171	0.367	0.171	0.00000
Masters degree	0.084	0.113	0.084	0.00000
Doctorate degree	0.011	0.007	0.011	0.00000
Never married/single	0.269	0.547	0.269	0.00000
Married	0.533	0.359	0.533	0.00000
Separated	0.023	0.015	0.023	−0.00000
Divorced	0.114	0.067	0.114	−0.00000
Widowed	0.061	0.012	0.061	−0.00000
New England	0.048	0.047	0.048	−0.00000
Middle Atlantic	0.134	0.132	0.134	0.00000
East North Central	0.150	0.151	0.150	−0.00000
West North Central	0.066	0.058	0.066	−0.00000
South Atlantic	0.196	0.239	0.196	0.00000
East South Central	0.060	0.063	0.060	−0.00000
West South Central	0.114	0.085	0.114	0.00000
Mountain	0.070	0.066	0.070	0.00000
Pacific	0.161	0.159	0.161	0.00000
Household income under $20K	0.134	0.148	0.134	−0.00000
Household income $20–30K	0.093	0.137	0.093	0.00000
Household income $30–40K	0.094	0.133	0.094	−0.00000
Household income $40–50K	0.090	0.121	0.090	−0.00000
Household income $50–60K	0.084	0.104	0.084	−0.00000
Household income $60–70K	0.076	0.080	0.076	0.00000
Household income $70–80K	0.067	0.073	0.067	0.00000
Household income $80–90K	0.058	0.049	0.058	−0.00000
Household income $90–100K	0.048	0.047	0.048	0.00000
Household income $100–110K	0.043	0.039	0.043	−0.00000
Household income $110–120K	0.034	0.012	0.034	0.00000
Household income $120–130K	0.029	0.016	0.029	0.00000
Household income $130–140K	0.023	0.007	0.023	0.00000
Household income $140–150K	0.019	0.007	0.019	−0.00000
Household income over $150K	0.109	0.028	0.109	0.00000

Notes: The ACS reference sample includes all individuals aged at least 18 years and not living in institutions.

### 0.3 Conditional logit

We used the conditional logit to model consumer responses to product attributes for cold medication. The conditional logit model can estimate how the attributes of a choice influence the probability of being chosen from among several alternatives. Specifically we modeled the probability of choice *j* out of *J* total choices as:

$$\begin{array}{c} p_{ij}=\mathrm{Pr}[y_{i}=j]=\frac{\exp\left(\beta_{P}P_{ij}+\sum_{n=1}^{N}\beta_{R_{n}}R_{ijn}\right)}{\sum_{k=1}^{J}\exp\left(\beta_{P}P_{ik}+\sum_{n=1}^{N}\beta_{R_{n}}R_{ikn}\right)}, \end{array}$$ (1)

where *p*_*ij*_ was the probability individual *i* makes choice *j*. Each choice was defined by a price given by *P*_*ij*_ and a set of restriction indicators each represented by *R*_*ijn*_. The impact of price on the probability of choice was captured by the *β*_*P*_ parameter and the impact of the *n* ^th^ restriction on choice was captured by the parameter *β*_*R*_*n*__. For choices involving prescription-only restrictions we included the self-reported copay amounts as part of the price when modeling the impact of price on choice in the conditional logit models. We recovered the willingness-to-accept a particular restriction from the ratio of the estimated restriction parameter to the price parameter, namely ${\hat{WTA}}_{R_{n}}=-{\hat{\beta }}_{Rn}/{\hat{\beta }}_{P}$. Standard errors for the willingness-to-accept estimates were calculated with the delta method [27]. We used robust standard errors that accounted for within-respondent error correlation. To simplify the choice modeling, we disregard choices that result in non-purchase. In order to test for potential heterogeneity in demand for decongestants between those with and without experience with the medicine, we estimated an alternative specification that restricted the population to respondents who had purchased pseudo decongestants in the last year.

## Results

Table 3 shows the results from the conditional logit model. Column 1 gives the impact of each product attribute (price or restriction) on the probability of choice. Column 2 displays willingness-to-accept each restriction. Negative values in Column 2 correspond to desirable product attributes that respondents would pay more for. The amount respondents would require to be willing to accept an undesirable product attribute is denoted with a positive value. We found respondents were willing to accept behind-the-counter restrictions in exchange for $9.68 on average. The willingness-to-accept a more burdensome prescription requirement was $14.17. Participants were willing-to-accept a substitute phenyl-based medication instead of a pseudo-based one for $4.09, which was less than the value participants placed on the behind-the-counter and prescription restrictions. Requiring identification did not significantly impact respondent purchase decisions, the coefficient was small in magnitude and statistically insignificant at the 5% level. Respondents valued imposing purchase limits as a positive attribute for which they were willing to accept price increases, though this counter-intuitive estimate was smaller in magnitude than the undesirable restrictions.

**Table 3:** Conditional logit models of cold medicine choice, baseline model and with demand interactions, with sampling weights.

*Attributes*	All respondents		Past pseudo. purchase	
	(1)	(2)	(3)	(4)
	Coefficients	WTA	Coefficients	WTA
Total price (dollars)	−0.10*** (0.01)		−0.10*** (0.02)	
Purchase experience				
Over-the-counter (reference)				
Behind-the-counter but no prescription	0.98*** (0.12)	9.68*** (1.35)	1.20*** (0.20)	12.43*** (2.38)
Require doctor’s prescription	1.44*** (0.24)	14.17*** (2.25)	1.48*** (0.37)	15.38*** (3.56)
Active ingredient				
Pseudoephedrine (reference)				
Phenylephrine	0.42*** (0.12)	4.09*** (1.24)	0.71*** (0.22)	7.35*** (2.26)
ID requirement				
No ID required (reference)				
ID required	−0.07 (0.09)	−0.65 (0.88)	−0.24** (0.11)	−2.54** (1.14)
Purchase limit				
No limit (reference)				
Any limit	−0.39*** (0.08)	−3.85*** (0.76)	−0.49*** (0.12)	−5.08*** (1.16)
*Specification*				
Pseudo R^2^	0.24		0.22	
N (respondents)	1,561		648	
N (choice sets)	12,603		5,168	
N (alternatives)	37,809		15,504	

Notes: Standard errors that account for arbitrary correlation of errors by respondent in parentheses. Standard errors for willingness-to-accept (WTA) estimates are calculated with the delta method. Stars indicate statistical significance:

**p < 0.01.

***p < 0.001.

The model in Columns 3 and 4 is restricted to respondents with recent experience with pseudo. Experienced pseudo purchasers comprised approximately 60% of our sample and placed slightly higher valuations on sales restrictions than the inexperienced survey respondents. Experienced pseudo users valued an active ingredient of phenyl over pseudo at $7.35 which was significantly higher than the $4.09 for the full sample.

## Discussion

We administered a choice experiment to assess willingness-to-accept inconvenience when purchasing pseudo-based nasal decongestants. We found respondents required $9.68 to accept behind-the-counter purchases, and $14.17 to accept prescription requirements. We also found consumers were willing to pay $4.09 for pseudo-based products compared to phenyl-based products.

Retail-level regulations requiring prescriptions for purchase or medication to be stored behind-the-counter may make it more difficult for illegal small-batch meth producers to obtain sufficient amounts of precursor needed to produce meth. But the regulations cannot discriminate between a consumer acquiring pseudo to treat cold symptoms and a consumer acquiring pseudo to manufacture meth, so the burden falls on both legitimate and illegitimate consumers.

There are several important limitations to our study. First, our choice experiment presented respondents with theoretical scenarios with different prices and restrictions. Previous studies have found that respondents do not necessarily pick the option with the maximum payoff [28]. Respondents in our study may be picking options without proper attention to price variables in our study. If respondents placed more weight on the prescription or other requirements in our experiment than they would have outside of the experimental setting, we would overestimate the willingness-to-accept these requirements.

Second, while both simple and complex experimental results from surveys conducted with Amazon Mechanical Turk have been cross-validated with results from populations derived in other settings [24, 29], some differences between Mechanical Turk workers and the general popultion have been identified. Amazon Mechanical Turk workers are generally less susceptible to subtly induced biases in survey questionnaire wording or misleading questions than are other reference populations [30]. Our survey was kept simple to mitigate such concerns when extrapolating our results to the general population.

We estimated weights to match the demographics of our survey respondents to those of the United States population. It is possible, however, that respondents may differ in unobservable characteristics. All Mechanical Turk workers have access to a computer and are comfortable inputting their Social Security Number over the Internet. We view this work as an important methodological first step, and encourage further studies to cross validate the results in other populations.

The willingness-to-accept estimates can be multiplied by the number of boxes to estimate the annually recurring impact of these restrictions on consumer welfare if all states were to implement behind-the-counter or prescription requirements. While no national estimates of boxes of pseudoephedrine sales exist, annual sales of pseudoephedrine by weight were estimated at 203,734 kg for 2010 [31]. A large box of pseudoephedrine contains 96 pills, and each pill contains 30 mg of pseudoephedrine. If all pseudoephedrine went into boxes with this configuration, there were 70,740,972 boxes sold nationwide in 2010. The DEA estimates 19.5% of retail, over-the-counter sales of pseudo were diverted to legitimate use [32]. Multiplying the estimated 56,946,482 boxes sold for legitimate use by the impacts on consumer welfare from our model, we estimate approximately $550 million in lost consumer welfare for behind-the-counter restrictions and $810 million in lost consumer welfare if prescriptions were required nationwide for pseudo purchases. These costs must be balanced against potential benefits from the restrictions that lower methamphetamine production and use.

Restricting access to medicines as a means of reducing the social costs of non-medical use can imply large inconvenience costs for legitimate consumers. Here we estimate these costs, although comparisons with the potential benefits of these restrictions need to be conducted on a case-by-case basis for each drug. This work has potential implications for estimating the benefits of making other medications available over-the-counter.

## Supporting Information

S1 Pseudo Orientation Orientation for survey participants to decongestant medications. (PDF)

S1 Dataset Dataset with survey responses to reproduce conditional logit model. (DTA)
