# Supplementary material for: Willingness-To-Accept Pharmaceutical Retail Inconvenience: Evidence from a Contingent Choice Experiment
Source: PLoS One. 2015 May 29;10(5):e0126790. doi: 10.1371/journal.pone.0126790 (PMC4449192; doi:10.1371/journal.pone.0126790)
Supplement: S1 Pseudo Orientation — (PDF) [file pone.0126790.s001.pdf]

Nasal decongestants are often used to treat nasal congestion and allergy symptoms.

These chemicals can be found in many over-the-counter and behind-the-counter cold medications such as Sudafed PE and Sudafed Pain and Pressure.

Common nasal decongestants use one of two primary active ingredients: **pseudoephedrine** and **phenylephrine**.

- There are some notable differences between these two types of chemical decongestants.
- On a molecular level, these chemicals differ in how much is metabolized by the body and which receptors are affected.
- Only 38% of phenylephrine is absorbed for effective use by the body compared with 100% for pseudoephedrine.
- Pseudoephedrine is a stimulant that releases adrenaline, whereas phenylephrine does not have this effect.

The following three images show decongestant medicines that contain **pseudoephedrine**:

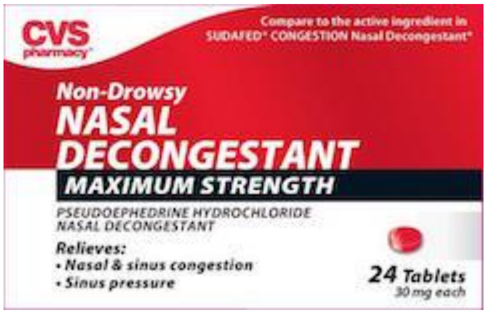

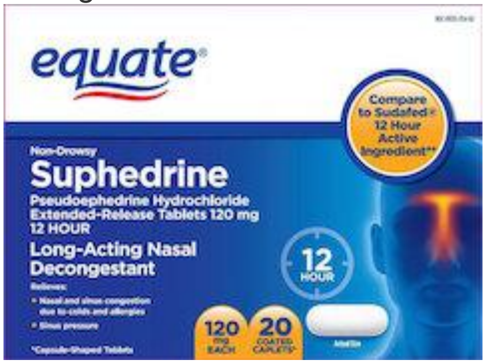

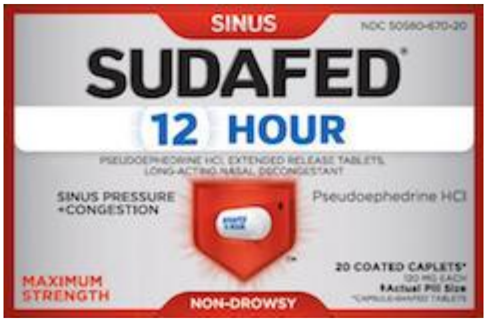

The following three images show decongestant medicines that contain **phenylephrine (not pseudoephedrine)**:

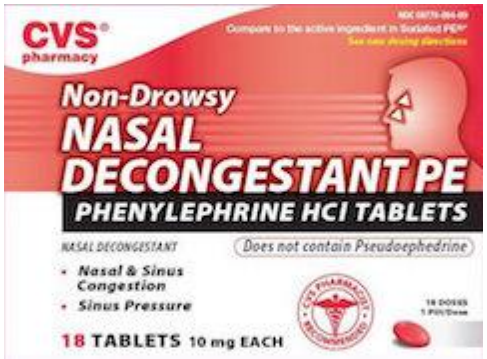

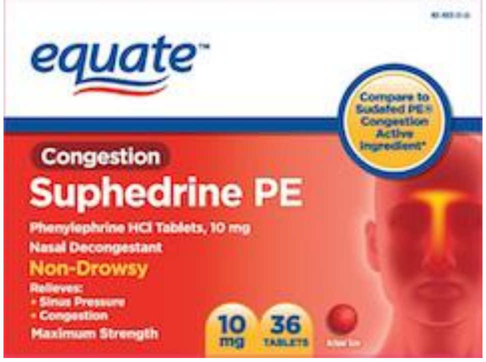

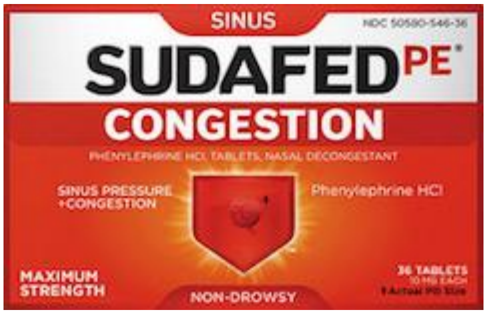

<< >>
